# Identification of Hub Genes Associated With Melanoma Development by Comprehensive Bioinformatics Analysis

**DOI:** 10.3389/fonc.2021.621430

**Published:** 2021-04-12

**Authors:** Jie Jiang, Chong Liu, Guoyong Xu, Tuo Liang, Chaojie Yu, Shian Liao, Zide Zhang, Zhaojun Lu, Zequn Wang, Jiarui Chen, Tianyou Chen, Hao Li, Xinli Zhan

**Affiliations:** Spinal Orthopedic Ward, The First Clinical Affiliated Hospital of Guangxi Medical University, Nanning, China

**Keywords:** melanoma, weighted gene co-expression network analysis, differential expression gene analysis, biomarker, predict prognosis, immunohistochemistry

## Abstract

**Introduction:**

This study aimed to identify important genes associated with melanoma to further develop new target gene therapies and analyze their significance concerning prognosis.

**Materials and methods:**

Gene expression data for melanoma and normal tissue were downloaded from three databases. Differentially co-expressed genes were identified by WGCNA and DEGs analysis. These genes were subjected to GO, and KEGG enrichment analysis and construction of the PPI visualized with Cytoscape and screened for the top 10 Hub genes using CytoHubba. We validated the Hub gene’s protein levels with an immunohistochemical assay to confirm the accuracy of our analysis.

**Results:**

A total of 435 differentially co-expressed genes were obtained. Survival curves showed that high expression of FOXM1,\ EXO1, KIF20A, TPX2, and CDC20 in melanoma patients with 5 of the top 10 hub genes was associated with reduced overall survival (OS). Immunohistochemistry showed that all five genes were expressed at higher protein levels in melanoma than in paracancerous tissues.

**Conclusion:**

FOXM1, EXO1, KIF20A, TPX2, and CDC20 are prognosis-associated core genes of melanoma, and their high expression correlates with the low prognosis of melanoma patients and can be used as biomarkers for melanoma diagnosis, treatment, and prognosis prediction.

## Introduction

Melanoma, a highly malignant tumor originating from melanocytes, most commonly occurs in the skin and can evolve from a congenital benign cell nevus or develop from a dysplastic nevus. According to a 2014 article on the epidemiology of melanoma: men are about 1.5 times more likely to develop melanoma than women; the development of malignant melanoma may be associated with changes in external environmental factors (e.g., UV exposure) (1). In the past few years, the primary means of treating melanoma have included: surgery, medication, and radiation therapy (2). A review of the literature on microsurgery versus extensive local excision for melanoma showed that after controlling for potential confounding variables, patients treated for melanoma with microsurgery were more likely to be alive at five years than those treated for melanoma with extensive local excision (3). Despite the many treatment options for melanoma, patient survival is still limited.

In recent years, with the rapid development of bioinformatics technology, bioinformatics has become increasingly popular for studying the molecular mechanisms of diseases and discovering disease-specific biomarkers that are increasingly being used to diagnose and treat diseases accurately (4–6). Weighted gene co-expression network analysis (WGCNA), one of the bioinformatics analysis methods, describes co-expression patterns between genes in microarray samples, providing new insights for predicting the function of co-expressed genes and the development of diseases (7). Differentially expressed genes(DEGs) are widely used in cancer research and are excellent cancer research methods (8–10). It provides a genomics-based method for discovering changes in gene expression levels between experimental and control groups (11). We could look for potential disease-related biomarkers in genes with significant differences in gene expression. Thus, we have combined the two approaches by combining genes from relevant modules obtained by the WGCNA method with differentially expressed genes to enhance the discrimination of genes that are expected to be candidate markers of disease.

In this study, in order to obtain melanoma hub genes, we analyzed melanoma mRNA expression data downloaded from the UCSC database, GTEx database, and GEO database by WGCNA method and differential expression gene method. We further verified our analysis’s accuracy and reliability by GO enrichment analysis, KEGG pathway enrichment analysis, protein-protein functional interaction network (PPI), survival analysis, and immunohistochemistry experiments to validate the results obtained from the analysis.

## Materials and Methods

### Data Download

Gene expression data for melanoma were downloaded from UCSC Xena (http://xena.ucsc.edu/) and the GEO database (https://www.ncbi.nlm.nih.gov/gds/). Furthermore, we downloaded all the data and corresponding clinical information on melanoma from the UCSC Xena database free of charge. Data downloaded from the UCSC Xena database contained 471 melanoma samples; gene expression data for 813 normal skin samples were downloaded from the GTEx database (https://www.gtexportal.org/home/); the number of melanoma cases for which all clinical information was complete was 322. In total, 55,188 genes were included in our acceptance of our subsequent analysis. On the other hand, we downloaded melanoma sample GSE3189 from the GEO database, a dataset containing 45 melanoma samples, 18 nevus samples, and 7 normal skin samples (12). We removed 18 moles from the sample. This dataset was studied using the platform GPL96 [HG-U133A] Affymetrix Human Genome U133A Array. Based on the manufacturer’s annotation files, we converted the probe files into gene symbols and removed duplicate probes, all expression data were transformed by log2, and the data were standardized. Eventually, a total of 12,549 genes were included for our subsequent analysis.

### Using WGCNA to Identify Key Co-Expression Modules

WGCNA is a framework for establishing and analyzing weighted gene co-expression networks and is a widely used bioinformatics analysis method that uses the interrelationship between two variables to study biological networks. In this study, all statistical analysis operations were performed based on R (x64 version 4.0.2). We used the R package (WGCNA) to construct a gene co-expression network from the gene expression data of melanoma from UCSC Xena and the gene expression data of melanoma data of GSE3189 from the GEO database (7). To construct the scale-free network, we use the R command: softPower =sft$powerEstimate, which commands R to automatically select the optimal power value, which ends up with a soft threshold of 12 for the UCSC Xena database and 5 for the GEO database. We then construct the adjacency matrix by the following formula: a_ij_ = power (*S_ij_*, β) = |*S_ij_*|^β (a_ij_ denotes the adjacency matrix between gene i and gene j; *S_ij_* denotes a similarity matrix completed by Pearson correlations for all gene pairs.; β denotes the soft threshold). Subsequently, we calculated the degree of dissimilarity between the nodes and converted the adjacency matrix into a TOM matrix. After that, we identified gene networks/modules using a dynamic shear tree algorithm. We correlated previously computed module features with clinical features to investigate the co-expression network’s functional modules further. As a result, the modules that were subsequently selected were closely correlated with clinical features and were selected by us for subsequent analyses. More detailed methods have been elucidated in detail by previous researchers (7).

### Identification of Differentially Expressed Genes and Selection of Significantly Expressed Modules

We used the R package (Limma) to perform differentially expressed genes (DEGs) analysis on gene expression data from both databases (13). To identify DEGs between melanoma and normal tissues, we performed the differential analysis of gene expression matrices from the two databases using the limma package, respectively. Cut off value was set to |logFC|>=1, adjusted P-value<0.05. We then used the R package (pheatmap) to construct heat maps of the DEGs filtered from the two databases; the R package (ggplot2) to plot the DEGs volcanoes. Next, co-expression genes extracted from the co-expression network overlapped with DEGs were used to identify potential prognostic genes. The overlapping portions of genes were used for subsequent analysis using the R package (VeenDiagram) (14)to visualize and plot the genes’ overlapping portions into Veen diagrams.

### GO Enrichment Analysis and KEGG Pathway Enrichment Analysis of Target Genes

To explore the overlapping parts of Gene Ontology (GO) (15) and KEGG pathway enrichment analysis (16), we used the R package (clusterProfiler, org.Hs.eg.db, enrichplot, ggplot2) (17) for analysis and visualization. The cut off value is set to p-value<0.05. The GO enrichment analysis consists of three main panels, namely Cellular component (CC), Biological process (BP), and Molecular function (MF). KEGG pathway enrichment analysis was mainly enriched in Melanoma, Transcriptional misregulation in cancer.

### Construction of Protein-Protein Interaction Networks and Screening of Hub Genes

We used the STRING (https://string-db.org/) (18) online database to construct protein-protein interaction (PPI) networks for overlapping partial genes. Subsequently, the resulting PPI network is imported into Cytoscape (v3.8.0) to visualize the PPI. We used a plugin in Cytoscape (CytoHubba) (19) to find Hub genes in these genes. We used the MCC algorithm, and the top 10 genes obtained to the MCC algorithm were used as the central genes for our study.

### The Relationship Between Hub Genes and Prognosis, and Their Expression in Various Subgroups

In this study, to verify Hub genes’ reliability, we used information from patients whose clinical information was complete for survival analysis. All patients were divided into two groups based on Hub genes’ median expression value, with patients greater than or equal to the median value assigned to the high expression group and patients less than the median value assigned to the low expression group. We plotted the Kaplan-Meier univariate survival analysis of overall survival (OS) using the R package (survival, survminer) for the top 10 hub genes. To further explore the effect of Hub genes on prognosis, we analyzed the relationship between five Hub genes and survival status and survival time using multivariate COX regression and constructed a prognostic model. We calculated the risk value of each patient and included patients with higher than average risk values in the high-risk group and those with lower or equal risk values in the low-risk group, and subsequently plotted Kaplan-Meier curves according to the high-risk and low-risk groups. Subsequently, we analyzed the top 10 Hub genes concerning disease-free survival using the online database GEPIA2 (http://gepia.cancer-pku.cn/). After that, we explored the differential expression of Hub genes in cancer and normal tissues, plotting each Hub genes’ expression levels in different subgroups between cancer and normal tissues as a box line graph.

### Immunohistochemistry

We used tumor sections and normal skin sections of melanoma patients who underwent surgical treatment at the First Clinical Affiliated Hospital of Guangxi Medical University for immunohistological studies, which were approved by the Ethics Department of the First Clinical Affiliated Hospital of Guangxi Medical University and conformed to the World Medical Association Declaration of Helsinki. We performed immunohistological analysis of six pairs (melanoma and normal skin) of pathological sections for each gene. Immunohistochemical staining of formalin-fixed melanoma tissue samples and paraffin-embedded and paracancerous tissue samples were performed. The FOXM1 and TPX2 antibodies for immunohistochemical staining were purchased from the Abcam; the KIF20A antibody was purchased from the Bioss; the CDC20 antibody was purchased from the Proteintech; the EXO1 antibody was purchased from the Abclonal. After removing paraffin, hydration, and sealing, the specimens were mixed with anti-FOXM1, KIF20A, TPX2, CDC20, and EXO1 and incubated overnight at 4°C (dilution ratios of 1:250, 1:200, 1:4000, 1:300, 1:100, respectively). Finally, in order to calculate the positivity rate of immunohistology images more precisely, we performed statistical analysis of images from immunohistology studies using Image J software. We then performed statistical analysis of the immunohistological positive rate for melanoma and the immunohistological positive rate for normal skin samples using the paired sample mean t-test in IBM SPSS Statistics 25 software. Finally, we use GraphPad Prism 8 to visualize the statistical results.

## Results

### Construction of Weighted Gene Co-Expression Modules

**Figure 1:**
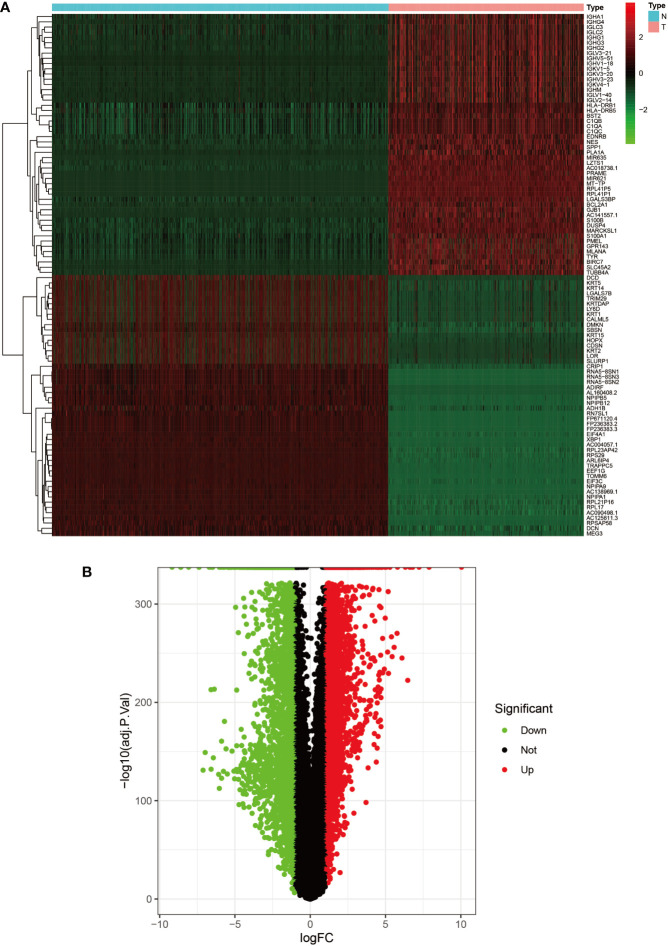
Heat map and volcano map of DEGs from the UCSC Xena database. **(A)** Heat map of the top 50 upregulated and top 50 down-regulated DEGs obtained from the identification. The red part indicates upregulated genes, and the green part indicates down-regulated genes. **(B)** Volcano plot with cut off value set to |logFC|>1,P-value<0.05. Red dots indicate upregulated genes, green dots indicate down-regulated genes, and black represents non-significant genes.

**Figure 2:**
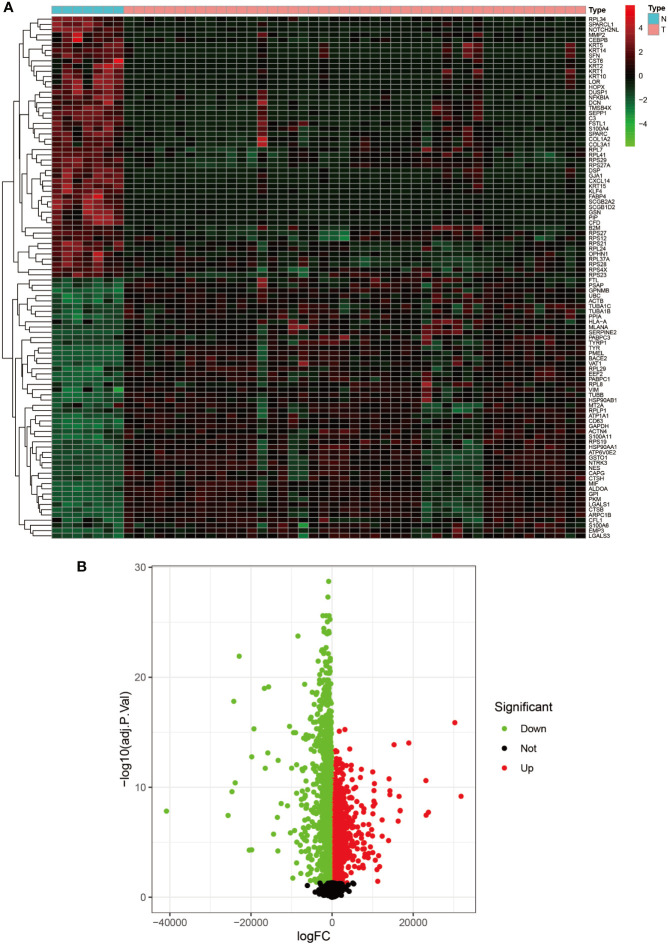
Heat map and volcano map of GSE3189. **(A)** Heat map of the top 50 upregulated and top 50 down-regulated DEGs obtained from the identification. The red part indicates upregulated genes, and the green part indicates down-regulated genes. **(B)** Volcano plot with cut off value set to |logFC|>1,P-value<0.05. Red dots indicate upregulated genes, green dots indicate down-regulated genes, and black represents non-significant genes.

To identify modules associated with prognosis in patients with melanoma, we performed gene co-expression network analysis of gene expression matrices from the UCSC Xena database and GSE3189 using the WGCNA package. There are six co-expression modules constructed from UCSC Xena database expression data (**Figure 1A**) and seven co-expression modules constructed from GSE3189 (**Figure 2A**), not including the grey module cluster to which it is assigned. We developed a heat map module features’ relationship, which was used to assess each co-expression module’s relationship with two clinical features (normal and cancer). The two modules’ features are shown in **Figure 1B** and **Figure 2B**. From the pictures, we can find that the highest correlation between the magenta module in UCSC Xena and the blue module in GSE3189 and normal organization (magenta module: r=-0.98, P-value<1e-200; blue module: r=-0.96, P-value<1e-200). Therefore, we extracted the genes from these two modules to further dig deeper into the useful information in them.

### Identification of DEGs and Gene Identification With Co-Expression Modules

**Figure 3:**
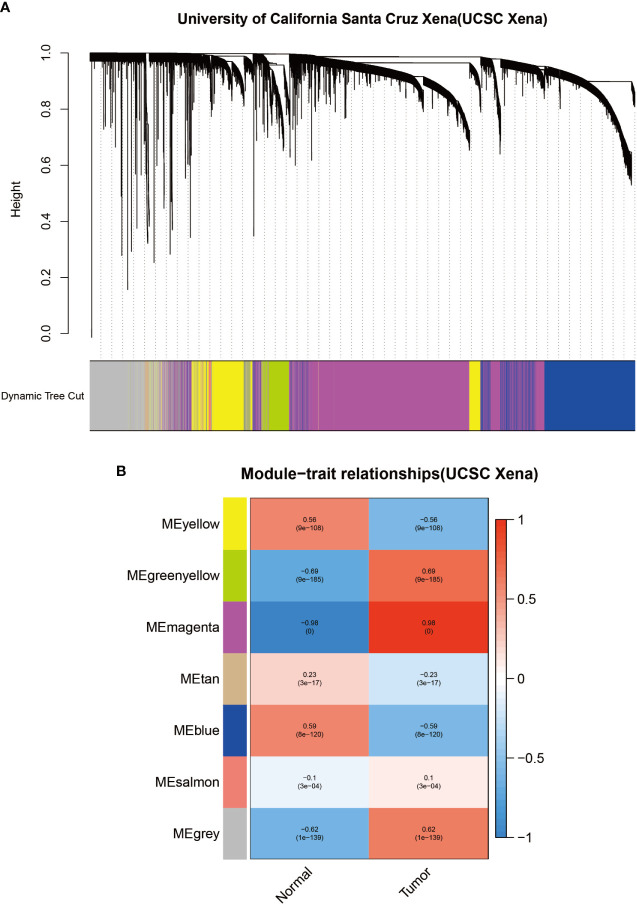
Identification of modules associated with clinical information in the UCSC Xena database. **(A)** Clustered tree diagram of co-expression network modules sorted by gene-level clustering of matrices obtained by Equation 1-TOM. Each color represents a different co-expressed gene. **(B)** Relationship diagram of the features of the modules. Each row corresponds to a color module, and each column corresponds to a clinical feature (normal and cancer). Each cell contains the correlation and P-value of the corresponding module.

**Figure 4:**
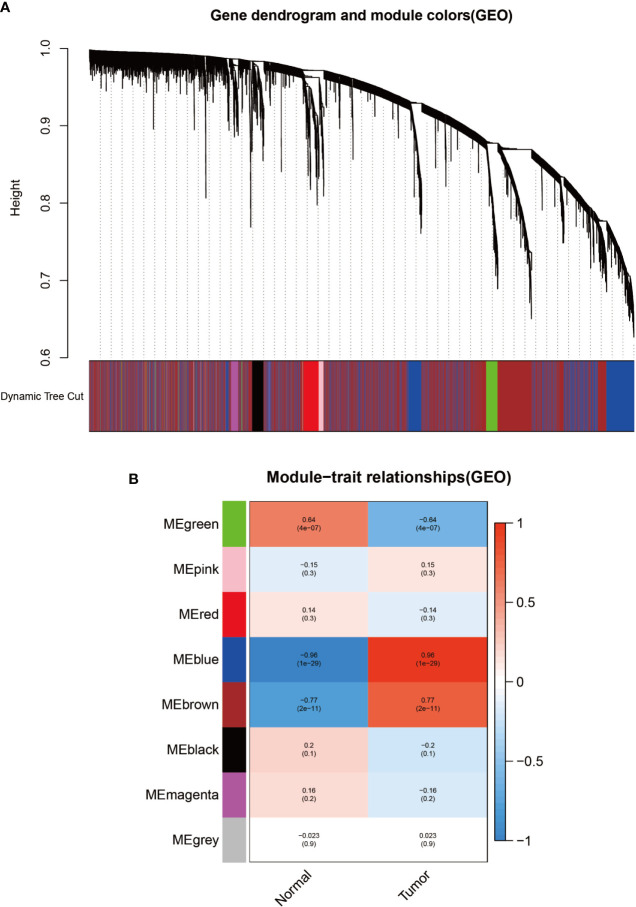
Identification of modules related to clinical information in the GSE3189 database. **(A)** Clustered tree diagram of co-expression network modules sorted by gene-level clustering of matrices obtained by Equation 1-TOM. Each color represents a different co-expressed gene. **(B)** Relationship diagram of the features of the modules. Each row corresponds to a color module, and each column corresponds to a clinical feature (normal and cancer). Each cell contains the correlation and P-value of the corresponding module.

**Figure 5:**
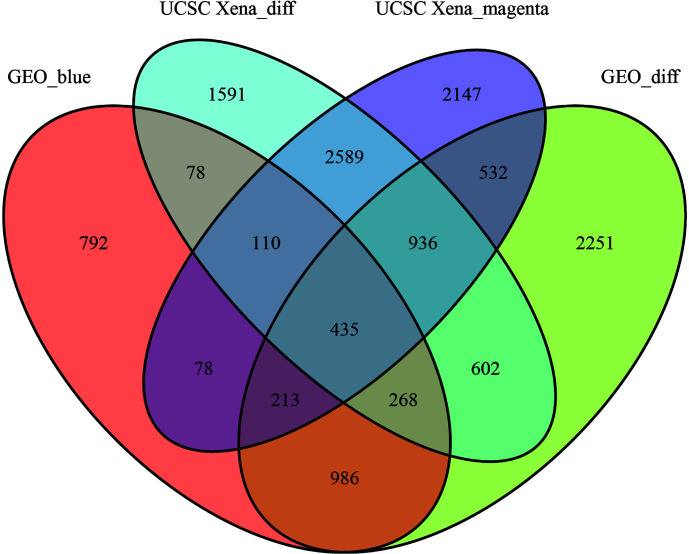
The veen plots between DEGs and co-expression modules. Geo_blue indicates the most significant module identified by WGCNA analysis of GSE3189; UCSC Xena_diff indicates DEGs identified by the UCSC Xena database; UCSC Xena_magenta indicates DEGs identified by WGCNA analysis The most significant modules out; GEO_diff indicates DEGs identified by GSE3189.

We set the cut-off value for DEGs as |logFC|>=1, adjusted P-value<0.05, and a total of 6609 DEGs were identified in the UCSC Xena dataset; heat maps and volcanoes of DEGs are shown in **Figure 3**; 6223 DEGs were identified in the GSE3198 dataset; heat maps and volcanoes of differential genes See **Figure 4**. We present the top 100 differentially expressed genes calculated from the GEO database in **Table 1**; the top 100 differentially expressed genes calculated from the UCSC Xena database are presented in **Table 2**. Genes from the magenta module in UCSC Xena and genes from the blue module in GSE3189, as well as DEGs from both databases, yielded a total of 435 overlapping genes extracted for subsequent analysis (**Figure 5**).

**Table 1:** Top 100 differentially expressed genes from UCSC Xena database differential expression analysis.

id	logFC	AveExpr	t	P. Value	adj. P. Val	B
HSD11B2	-845.579	176.0964	-29.2765	1.53E-33	1.92E-29	-4.0536
BCAM	-1017.36	323.9158	-26.9444	8.18E-32	5.13E-28	-4.0592
METTL7A	-2269.49	605.8938	-24.4868	7.60E-30	2.55E-26	-4.06674
LMOD1	-734.009	145.4836	-24.383	9.28E-30	2.55E-26	-4.06711
PLLP	-1437.35	434.1048	-24.3356	1.02E-29	2.55E-26	-4.06728
C1orf116	-494.735	91.70493	-23.8478	2.63E-29	5.50E-26	-4.06905
CDC20	389.965	482.6639	4.868726	1.13E-05	5.12E-05	-4.42177
PDZD2	-1159.37	213.1139	-23.5115	5.11E-29	9.16E-26	-4.07034
NFIB	-2039.56	544.4306	-22.7616	2.31E-28	3.63E-25	-4.07338
RAI2	-816.532	222.9485	-22.4094	4.77E-28	6.66E-25	-4.07491
SOD3	-1970.04	667.2363	-22.299	6.00E-28	7.53E-25	-4.0754
CLDN8	-739.437	164.3889	-22.1417	8.33E-28	9.50E-25	-4.07611
EPB41L4B	-1068.95	305.8102	-22.0679	9.72E-28	1.02E-24	-4.07644
TCF7L2	-1366.23	604.587	-22.0259	1.06E-27	1.02E-24	-4.07664
COBL	-1201.85	249.6083	-21.9426	1.26E-27	1.13E-24	-4.07702
NOTCH2NL	-8409.06	2198.169	-21.6906	2.15E-27	1.80E-24	-4.07822
DDAH1	-1470.08	560.094	-20.5529	2.54E-26	2.00E-23	-4.08409
PTPRK	-2420.67	866.2715	-20.3232	4.24E-26	3.13E-23	-4.08537
AKR1C3	-2026.43	340.9329	-20.173	5.94E-26	3.95E-23	-4.08624
BLCAP	-2587.68	2066.668	-20.1707	5.98E-26	3.95E-23	-4.08625
MKL2	-2073.3	890.3276	-19.8971	1.11E-25	6.96E-23	-4.08786
FOXC1	-924.752	352.5952	-19.8629	1.20E-25	7.17E-23	-4.08807
SCGB2A2	-22896.1	3285.843	-19.6068	2.16E-25	1.23E-22	-4.08964
HLF	-432.825	163.8867	-19.397	3.50E-25	1.91E-22	-4.09096
GPRC5C	-452.72	116.5967	-19.1655	6.00E-25	3.14E-22	-4.09247
TPM1	-3431.4	1077.406	-19.1101	6.84E-25	3.43E-22	-4.09284
INHBB	-1629.71	542.9868	-19.015	8.55E-25	4.03E-22	-4.09347
KCNK5	-1100.18	338.3407	-19.0084	8.68E-25	4.03E-22	-4.09352
LPP	-1662.54	1114.69	-18.9759	9.38E-25	4.20E-22	-4.09374
BAG1	-1512.62	963.4278	-18.63	2.13E-24	9.04E-22	-4.09613
BBOX1	-1291.96	255.0336	-18.6242	2.16E-24	9.04E-22	-4.09617
NEBL	-609.949	272.7083	-18.4247	3.49E-24	1.41E-21	-4.0976
SEMA3G	-2467.01	524.8589	-18.2258	5.65E-24	2.21E-21	-4.09907
AOX1	-462.531	149.6399	-18.1128	7.44E-24	2.83E-21	-4.09992
OSR2	-1470.39	407.5353	-17.9596	1.08E-23	4.00E-21	-4.1011
AFF1	-921.029	598.5215	-17.915	1.21E-23	4.30E-21	-4.10145
ANK3	-626.259	224.7039	-17.9063	1.23E-23	4.30E-21	-4.10151
TNS1	-3044.64	867.9854	-17.7824	1.68E-23	5.69E-21	-4.10249
KIF20A	549.666	571.9327	5.727454	5.48E-07	3.36E-06	-4.37847
RNASE4	-1188.56	335.7413	-17.3161	5.38E-23	1.77E-20	-4.10632
KRT23	-1993.11	437.5972	-17.3038	5.55E-23	1.77E-20	-4.10642
TPD52L1	-3095.22	669.0397	-17.297	5.64E-23	1.77E-20	-4.10648
SYBU	-920.434	364.3275	-17.2781	5.92E-23	1.81E-20	-4.10664
ADRB2	-1071.55	319.8041	-17.2099	7.04E-23	2.10E-20	-4.10722
FBXW12	-2471.35	1018.696	-17.0651	1.02E-22	2.97E-20	-4.10848
NXN	-3120.53	896.8017	-16.9564	1.34E-22	3.83E-20	-4.10944
SLK	-1140.23	736.8274	-16.9376	1.41E-22	3.93E-20	-4.10961
NPR1	-306.481	115.5731	-16.9289	1.44E-22	3.93E-20	-4.10969
KLF4	-6760.93	1402.794	-16.8796	1.63E-22	4.36E-20	-4.11013
TACC2	-1660.96	469.7259	-16.6594	2.89E-22	7.53E-20	-4.11214
CFD	-15658.5	2463.293	-16.6522	2.94E-22	7.53E-20	-4.11221
MANSC1	-749.192	222.4662	-16.556	3.78E-22	9.47E-20	-4.11311
SCGB1D2	-16696.7	2299.639	-16.5155	4.20E-22	1.03E-19	-4.11349
CYP4F12	-834.366	226.1397	-16.4526	4.94E-22	1.19E-19	-4.11409
ARHGAP29	-1972.3	562.7853	-16.4261	5.30E-22	1.23E-19	-4.11435
PAPD7	-1521.75	1286.389	-16.426	5.30E-22	1.23E-19	-4.11435
HOXA5	-1120.97	339.1495	-16.1654	1.05E-21	2.34E-19	-4.1169
TPX2	844.8853	941.0447	6.308941	6.73E-08	5.11E-07	-4.35103
AKR1C1	-4886.37	1050.501	-16.1645	1.06E-21	2.34E-19	-4.11691
PGRMC2	-789.323	518.6895	-16.1623	1.06E-21	2.34E-19	-4.11693
DEFB1	-3728.94	773.1849	-16.1416	1.12E-21	2.43E-19	-4.11714
NISCH	-3781.98	2154.822	-16.1158	1.20E-21	2.55E-19	-4.1174
PID1	-660.945	181.8225	-16.0308	1.51E-21	3.15E-19	-4.11826
ECHDC2	-2053.82	566.7756	-16.0213	1.54E-21	3.18E-19	-4.11835
ZNF721	-3930.63	1696.277	-15.9616	1.81E-21	3.66E-19	-4.11896
FZD10	-935.976	244.2876	-15.9546	1.84E-21	3.67E-19	-4.11904
FOXM1	407.8747	439.4756	4.447452	4.73E-05	0.000185	-4.44386
MGST2	-1763.19	647.3899	-15.9227	2.01E-21	3.94E-19	-4.11937
CLDN5	-1893.35	403.8225	-15.9111	2.07E-21	4.00E-19	-4.11948
PGF	-1363.34	863.4405	-15.8693	2.32E-21	4.40E-19	-4.11992
MYO5C	-2220.87	777.6688	-15.8323	2.56E-21	4.79E-19	-4.12031
RIOK3	-807.68	826.6529	-15.7848	2.91E-21	5.37E-19	-4.1208
SLIT3	-750.592	281.9049	-15.5706	5.19E-21	9.43E-19	-4.1231
DKFZP586I1420	-1528.75	783.6597	-15.5415	5.61E-21	1.01E-18	-4.12342
IRF6	-2781.83	874.8339	-15.4857	6.54E-21	1.15E-18	-4.12403
KLF2	-2970.81	1606.61	-15.4511	7.18E-21	1.25E-18	-4.12441
CA6	-3085.23	529.0964	-15.4115	8.00E-21	1.38E-18	-4.12485
EMX2	-455.309	100.5197	-15.3847	8.61E-21	1.46E-18	-4.12515
CXCL14	-24233.7	4778.02	-15.3585	9.25E-21	1.55E-18	-4.12544
PPAP2A	-2079.6	1113.894	-15.2372	1.29E-20	2.13E-18	-4.12682
MAST4	-2097.42	497.6463	-15.2144	1.37E-20	2.24E-18	-4.12708
CARD10	-205.013	117.4037	-15.1947	1.45E-20	2.33E-18	-4.12731
LAMA3	-1212.8	241.0786	-15.1253	1.76E-20	2.79E-18	-4.12811
TACSTD2	-4413.24	865.4007	-15.1017	1.88E-20	2.94E-18	-4.12838
LIMS2	-1426.29	640.6591	-15.077	2.01E-20	3.11E-18	-4.12867
FAM117A	-1093.53	570.1012	-15.0156	2.38E-20	3.65E-18	-4.1294
EXO1	205.6559	242.1595	5.043051	6.17E-06	2.97E-05	-4.41276
KRT18	-2757.63	602.0107	-14.9803	2.63E-20	3.97E-18	-4.12981
MYH11	-3199.73	525.7094	-14.9037	3.26E-20	4.86E-18	-4.13073
AZGP1	-2910.91	538.8767	-14.6937	5.87E-20	8.66E-18	-4.13329
KIAA0485	-632.996	214.483	-14.6163	7.30E-20	1.07E-17	-4.13426
PCNXL2	-186.091	153.767	-14.548	8.86E-20	1.28E-17	-4.13512
ATP6V0A4	-650.17	187.6908	-14.4758	1.09E-19	1.55E-17	-4.13604
PPP1CB	-868.475	534.1197	-14.4146	1.29E-19	1.82E-17	-4.13683
VWF	-3563.29	1541.653	-14.3503	1.56E-19	2.17E-17	-4.13767
SLC24A3	-519.411	131.0307	-14.3207	1.69E-19	2.33E-17	-4.13806
CHRDL1	-3642.25	614.9238	-14.3008	1.79E-19	2.44E-17	-4.13832
EFS	-860.828	247.5087	-14.284	1.88E-19	2.54E-17	-4.13854
N4BP2L2	-1182.53	803.6723	-14.2559	2.04E-19	2.72E-17	-4.13891
MAP3K4	-1498.72	670.2975	-14.2228	2.24E-19	2.96E-17	-4.13935

**Table 2:** Top 100 differentially expressed genes from GEO database differential expression analysis.

id	logFC	AveExpr	t	P. Value	adj. P. Val	B
TOMM6	-6.70878	4.247851	-441.582	0	0	3210.479
EEF1G	-9.17482	6.273636	-358.094	0	0	2946.723
ARL6IP4	-5.34494	4.568667	-270.314	0	0	2593.078
U2AF1	-4.89304	3.189738	-267.824	0	0	2581.47
KIF20A	2.257505	2.184619	35.43105	2.64E-192	3.28E-191	428.7177
TRAPPC5	-5.2377	3.597862	-265.289	0	0	2569.541
DDX47	-4.1377	3.218522	-263.144	0	0	2559.36
AC026464.4	-4.12231	2.667977	-234.28	0	0	2413.931
AC008894.2	-3.89785	2.492557	-231.702	0	0	2400.115
ZNF410	-3.7731	3.023446	-226.448	0	0	2371.487
AC138969.1	-6.58451	4.261407	-224.402	0	0	2360.166
EIF4A1	-6.5225	5.862644	-219.31	0	0	2331.554
XBP1	-5.49276	3.477893	-217.59	0	0	2321.745
RPS29	-5.7619	8.791745	-217.216	0	0	2319.602
RPL41P1	7.895229	2.907401	210.3463	0	0	2279.615
SARNP	-4.32075	3.616277	-209.23	0	0	2273.003
C17orf49	-4.48374	3.787316	-208.004	0	0	2265.695
AP003108.2	-3.86539	2.574211	-207.004	0	0	2259.706
NPIPA9	-6.47004	4.114255	-205.125	0	0	2248.382
U2AF1L5	-4.59973	2.953342	-204.651	0	0	2245.511
AL445363.3	-3.97251	2.527661	-204.223	0	0	2242.909
PLSCR3	-4.35472	3.215705	-204.113	0	0	2242.24
MT-TP	10.04972	3.69955	203.6833	0	0	2239.626
AC004057.1	-8.07281	5.336825	-200.98	0	0	2223.05
UBE2V1	-3.5256	4.398678	-200.156	0	0	2217.955
EIF3CL	-4.40254	3.018283	-194.219	0	0	2180.647
CHMP4A	-4.13699	4.114164	-192.739	0	0	2171.174
RPL23AP42	-5.9686	6.903515	-192.172	0	0	2167.53
RBM34	-3.73833	3.315043	-189.086	0	0	2147.518
FAM156A	-4.2274	2.753841	-188.932	0	0	2146.508
PSMC1	-3.54821	4.637263	-186.871	0	0	2132.96
AC234031.1	-4.1459	2.626985	-185.406	0	0	2123.242
EIF3C	-5.4449	5.199535	-181.198	0	0	2094.928
NDST2	-3.18778	2.770728	-179.958	0	0	2086.466
RPL41P5	5.796619	2.135216	178.8032	0	0	2078.539
POLR2J3	-4.77323	3.508843	-175.214	0	0	2053.594
NPIPA1	-5.2232	4.152355	-173.431	0	0	2041.021
MIR621	5.055425	1.860197	171.4897	0	0	2027.203
RPL17	-5.68075	7.892466	-170.602	0	0	2020.837
CBWD3	-3.53714	2.282208	-170.004	0	0	2016.528
KLC1	-3.77402	4.474801	-168.533	0	0	2005.876
FOXM1	2.234867	2.87717	39.80523	2.75E-226	4.47E-225	506.9552
AC090498.1	-5.26043	8.372091	-167.292	0	0	1996.823
PSMA6	-3.37821	4.795774	-166.672	0	0	1992.276
OVCA2	-4.03462	2.55463	-165.63	0	0	1984.594
MIA2	-2.42462	2.010625	-163.757	0	0	1970.685
SKP1	-3.17494	6.591382	-163.588	0	0	1969.42
RNASEK	-4.25662	5.849365	-163.016	0	0	1965.143
BBS1	-2.78795	2.33777	-162.105	0	0	1958.295
RPL36A	-4.99421	7.771035	-161.219	0	0	1951.603
FP236383.3	-8.60309	5.458479	-157.818	0	0	1925.6
FP236383.2	-8.60554	5.457578	-157.788	0	0	1925.369
FP671120.4	-8.58582	5.464812	-156.76	0	0	1917.402
ANKHD1	-4.26189	3.413317	-155.416	0	0	1906.921
ACAD11	-3.32657	2.373162	-155.294	0	0	1905.971
CBWD5	-4.00438	2.944129	-152.563	0	0	1884.402
EEF1D	-3.77282	6.967246	-150.509	0	0	1867.955
GTF2IP1	-4.45164	3.217918	-147.968	0	0	1847.323
SLX1A	-3.90342	2.487477	-147.257	0	0	1841.492
AARSD1	-2.72754	3.419285	-146.946	0	0	1838.933
LIMD1-AS1	-2.12395	1.48131	-146.44	0	0	1834.767
MEMO1	-2.96218	2.820104	-146.034	0	0	1831.403
RBM4	-3.22088	4.409497	-145.28	0	0	1825.147
TPX2	2.923316	3.337959	48.93582	1.21E-295	3.49E-294	666.6618
CBWD2	-2.6548	2.865867	-144.03	0	0	1814.718
ATRIP	-2.07223	1.378742	-143.514	0	0	1810.388
PSMA2	-3.10176	4.482894	-142.944	0	0	1805.587
CCZ1B	-3.28051	3.403163	-142.938	0	0	1805.538
CMC4	-2.51744	1.594191	-142.923	0	0	1805.411
TEN1	-4.06633	2.761896	-141.258	0	0	1791.289
INO80B-WBP1	-2.89703	1.857204	-141.116	0	0	1790.079
RPL39	-4.41911	8.980427	-141.005	0	0	1789.13
CBWD1	-3.30889	2.794308	-140.982	0	0	1788.929
TOP3B	-3.1789	2.223469	-140.981	0	0	1788.919
RPL21P16	-5.33883	6.932082	-139.741	0	0	1778.293
SNX15	-2.94194	2.13521	-139.636	0	0	1777.391
PDLIM2	-4.65201	4.800814	-139.272	0	0	1774.247
RPS15A	-3.63592	8.609921	-138.656	0	0	1768.919
SERF1B	-3.59774	2.582927	-137.965	0	0	1762.917
NPIPB5	-6.03868	4.198667	-137.514	0	0	1758.987
TREX1	-4.57687	2.897972	-135.122	0	0	1737.954
AC125611.3	-5.4162	3.434745	-134.031	0	0	1728.242
NDUFV2	-3.10642	4.693101	-133.939	0	0	1727.428
NPIPB3	-5.02126	3.613232	-133.427	0	0	1722.841
ARPC4-TTLL3	-3.05658	2.139033	-133.188	0	0	1720.697
ATXN3	-2.43806	2.558439	-132.717	0	0	1716.468
CDC20	2.434468	3.290483	34.57408	1.23E-185	1.45E-184	413.3673
WDR73	-2.31081	2.425118	-132.685	0	0	1716.175
AGAP5	-1.97576	1.318344	-131.963	0	0	1709.66
CCZ1	-3.07563	3.369659	-130.65	0	0	1697.731
NBPF10	-2.28028	1.696457	-129.814	0	0	1690.075
ASB3	-1.85253	1.913987	-128.674	0	0	1679.575
HNRNPCP2	3.389508	2.234573	127.7404	0	0	1670.918
RN7SL1	-5.42918	3.437632	-127.724	0	0	1670.761
OGFOD2	-2.30304	2.232653	-127.586	0	0	1669.478
H3F3A	-3.73636	6.047482	-126.989	0	0	1663.912
SLX1A-SULT1A3	-2.75166	1.760848	-125.534	0	0	1650.232
AC139256.2	-3.3025	2.974018	-125.191	0	0	1646.99
EXO1	1.960164	1.554693	42.1115	4.53E-244	8.56E-243	547.9027

### GO Enrichment Analysis of 435 Genes and KEGG Pathway Enrichment Analysis

**Figure 6:**
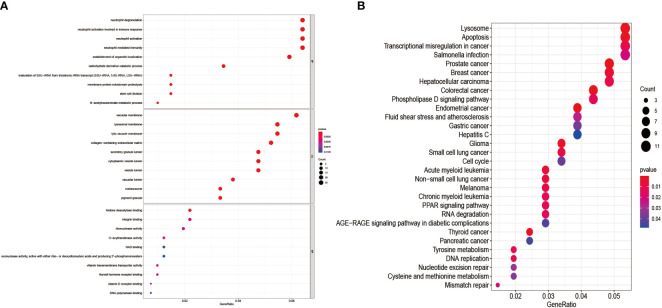
GO enrichment analysis and KEGG pathway enrichment analysis of DEGs. **(A)** shows the GO enrichment analysis of DEGs; **(B)** shows the DEGs’ KEGG pathway enrichment analysis.

We performed GO enrichment analysis, and KEGG pathway enrichment analysis further explores the 435 genes’ potential functions in the overlapping sections. We learned from the GO enrichment analysis that BP was primarily enriched in neutrophil degranulation and neutrophil activation involved in the immune response. CC was mainly enriched in vacuolar and lysosomal membranes. MF was mainly enriched in histone deacetylase binding and integrin-binding (**Figure 6A**). KEGG pathway enrichment analysis was mainly distributed in the melanoma, Transcriptional misregulation in cancer, and Mismatch repair pathways (**Figure 6B**).

### PPI Construction and Hub Gene Identification

**Figure 7:**
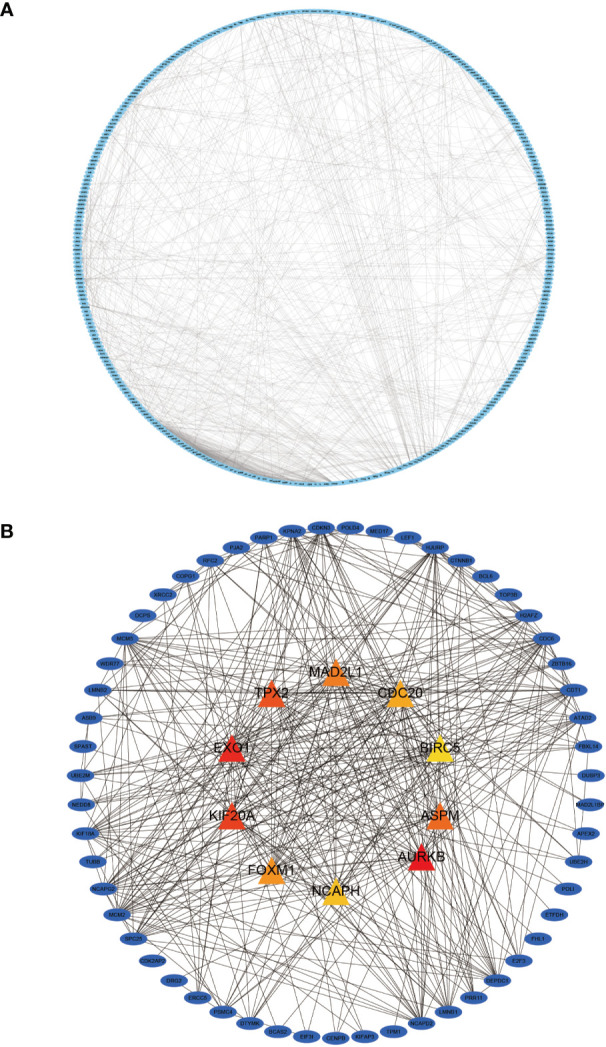
Protein-protein interaction (PPI) network diagram of DEGs and visualization of the Hub gene. **(A)** shows the PPI network diagram derived from the STRING input library; **(B)** shows the Hub gene identified by Cytohubba.

We imported 435 genes from the overlapping parts into the STRING online database to obtain the PPI network. Subsequently, the PPI network was imported into Cytoscape software to visualize the PPI (**Figure 7A**) using the MCC algorithm in the CytoHuba plugin to filter the top 10 Hub genes from the PPI network (**Figure 7B**). We ranked the top 10 Hub genes based on their MCC algorithm scores, which were Aurora Kinase B (AURKB), Exonuclease 1 (EXO1), Kinesin Family Member 20A (KIF20A), TPX2 Microtubule Nucleation Factor (TPX2), Assembly Factor For Spindle Microtubules(ASPM), Mitotic Arrest Deficient 2 Like 1 (MAD2L1), Forkhead Box M1(FOXM1), Cell Division Cycle 20(CDC20), Non-SMC Condensin I Complex Subunit H(NCAPH), Baculoviral IAP Repeat Containing 5(BIRC5).

### Prognostic Value of Hub Genes and Validation of Protein Expression

**Figure 8:**
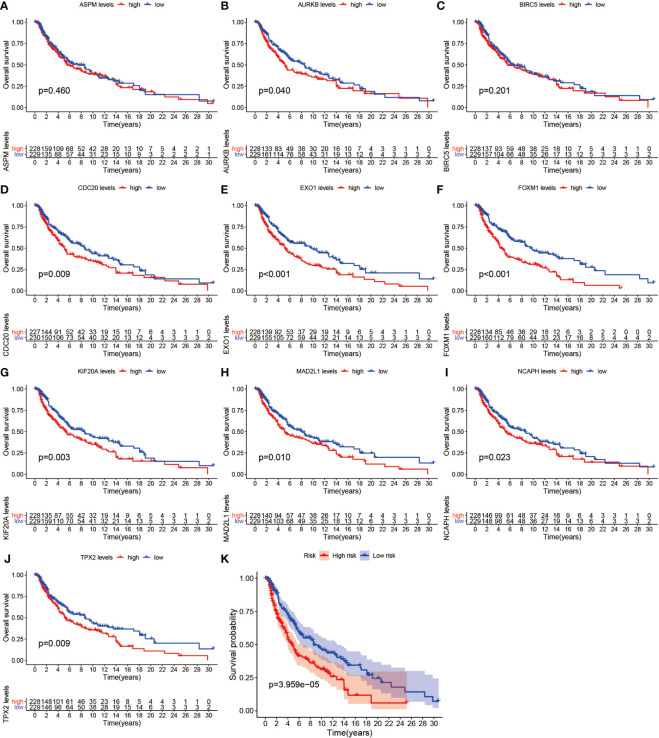
Survival analysis. **(A–J)** shows the survival curves of the first 10 Hub genes. **(K)** shows the survival curves for high-risk and low-risk based on the prognostic model.

**Figure 9:**
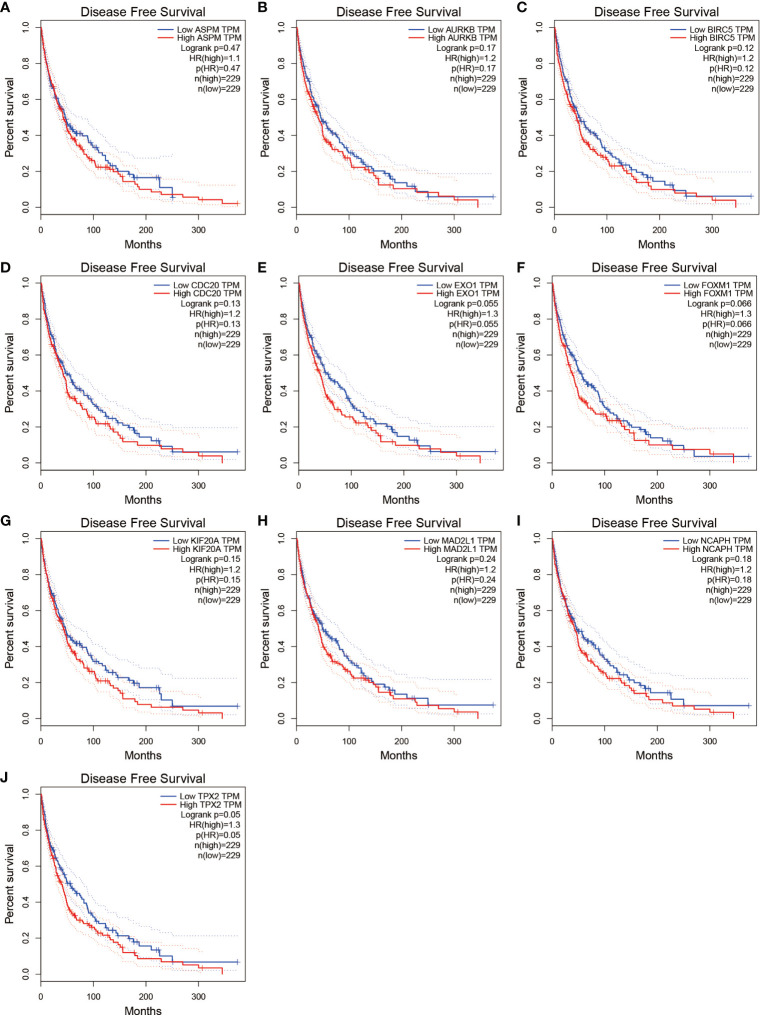
Disease-free survival for the top 10 Hub genes. **(A–J)** show the curves of disease-free survival based on the GEPIA2 database.

**Figure 10:**
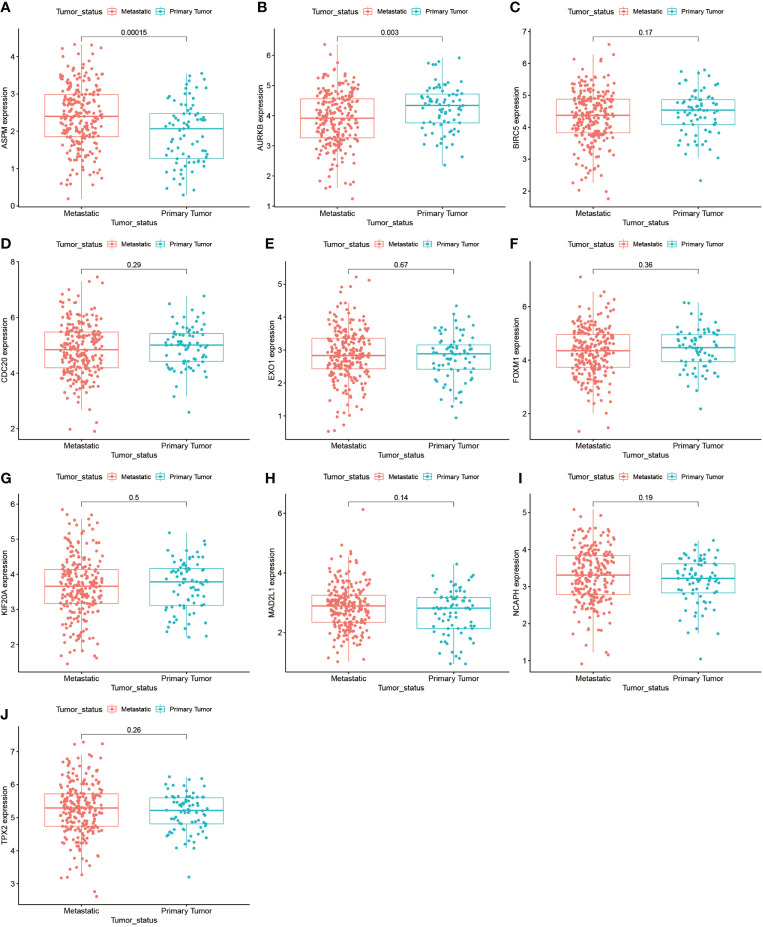
Gene expression of the top 10 Hub genes in the metastasis and primary tumor groups. **(A–J)** demonstrate the differences in gene expression of the top 10 Hub genes in the metastasis-bearing group and in the primary tumor group.

We further analyzed the top 10 Hub genes (AURKB, EXO1, KIF20A, TPX2, ASPM, MAD2L1, FOXM1, CDC20, NCAPH, BIRC5) that were screened by the CytoHubba plugin. We analyzed the relationship between the top 10 Hub genes and prognosis separately and plotted univariate survival curves for overall survival using the R package based on the Kaplan-Meier method. Kaplan-Meier analysis showed (**Figure 8**) that 5 of the top 10 Hub genes (FOXM1, EXO1, KIF20A, TPX2, CDC20) were significantly correlated with prognosis (p<0.01), and that the relationship between all five hub genes and the prognosis of melanoma patients was such that high expression of the gene was accompanied by low patient Prognosis. In addition, we analyzed the survival curves of the prognostic models constructed based on these five Hub genes, as shown in **Figure 8K**. The survival rate in the high-risk group was much lower than that in the low-risk group, and the difference was statistically significant (P-value < 0.05). We used the GEPIA2 database to predict disease-free survival for the top 10 Hub genes, and the predictions are shown in **Figure 9**. We divided all patients into two groups according to metastasis and primary status of the tumor. From **Figure 10A**, we found that the mean expression value of ASPM was significantly higher in the metastatic group than in the primary group (P-value<0.001); in **Figure 10B**, we found that the expression value of AURB was significantly higher in the primary group than in the metastatic group (P-value=0.003).

### Immunohistochemistry

**Figure 11:**
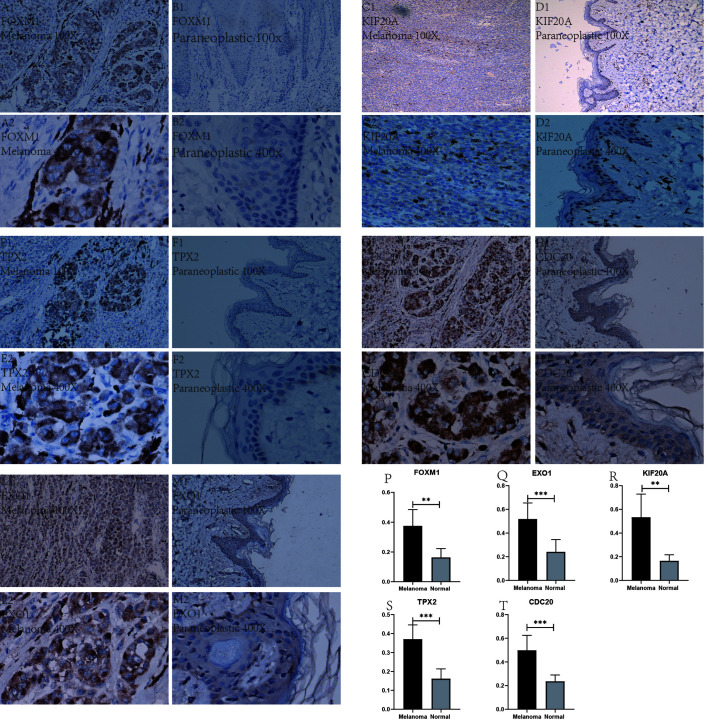
Immunohistochemical plots of the five Hub genes associated with prognosis and statistical analysis of the positivity rate. **(A1–M2)** show the protein expression of each gene in melanoma and in the paracancerous tissue. **(P–T)** shows the statistical analysis of the staining positivity rate for each gene in melanoma and in the paracancerous tissue. **representative P-value < 0.01, ***representative P-value < 0.001.

After a series of laboratory manipulations, we completed specific staining of all pathological tissue sections for labeled antibodies. All immunohistological images were observed under an inverted microscope and images were collected, and we compared the staining differences between melanoma specimens and paraneoplastic tissue specimens. We performed immunohistological staining analysis on a total of 60 pathological tissue sections of six pairs (melanoma tissue and normal skin tissue) for each gene, and the positive rate of each image was counted using Image J software. After IBM SPSS Statistics 25 paired sample mean t-test, finally, we visualized the statistical results using GraphPad Prism 8. After analysis of 60 immunohistochemical images, we found that these five Hub genes were significantly more abundantly expressed in melanoma than in paraneoplastic tissue. This result also further validates the accuracy and validity of our bioinformatics analysis. We selected from 60 immunohistochemical images the images with the most significant differences between these 5 Hub genes in melanoma and paracancerous tissue in **Figures 11A1–M2**. In addition, we counted the positive rate of immunohistological images of six pairs of pathological tissue sections for each antibody separately, and from **Figures 11P–T** we found that the positive rate of immunohistochemical staining for these five genes in melanoma was significantly higher than that in paraneoplastic tissue, and the difference was statistically significant.

## Discussion

Melanoma is associated with many factors, is more common in light-skinned races, and has a family history of occurrence. Although melanoma treatment has improved from before, the prognosis for melanoma patients is low due to the lack of precise molecular markers. Therefore, there is an urgent need to identify better and more accurate biomarkers to utilize in the prognosis, diagnosis, and treatment of melanoma. In our study, we used integrated bioinformatics to analyze a total of 435 critical genes with co-expression trends identified in the UCSC Xena, GTEx database, and the GSE3189 database. These genes were subjected to GO enrichment analysis based on the R package (clusterProfiler), mainly enriched in neutrophil activation involved in immune response, stem cell division, and melanosome. As early as 2011, it was noted that there might be a close relationship between neutrophil activation and cancer (20). Moreover, the relationship between the stem cell division and melanosome and cancer has also been reported in the literature (21, 22). Similarly, these genes were subjected to KEGG pathway enrichment analysis based on the R package (clusterProfiler), and the enrichment results showed that these genes were associated with various cancer pathways, including melanoma, endometrial cancer, prostate cancer, etc.; they were also enriched in transcriptional dysregulation pathways in cancer. In humans, dysregulation of genes such as cofactors and chromatin can lead to many diseases (23). These genes are even enriched in the melanoma pathway, suggesting that these genes are strongly associated with melanoma. Besides, we screened for the top 10 hub genes associated with melanoma based on how the MCC score was calculated for the CytoHubba plugin in Cytoscape. We also found that by analyzing the survival of melanoma patients corresponding to high and low expression of these genes, five of the top 10 hub genes were strongly associated with survival, and all showed that high expression of the genes was associated with a low prognosis in melanoma patients. Finally, we performed immunohistochemical analysis using the HPA database and showed that all four genes showed increased expression in melanoma tumor tissues, whereas their expression was not evident in normal tissues.

FOXM1, also known as Forkhead Box M1, is a gene that encodes a protein that is a transcriptional activator involved in cell proliferation. FOXM1 acts downstream of the PI3K-AKT pathway, the Ras-ERK pathway, the JNK/p38MAPK signaling cascade and is essential for cell proliferation, differentiation, senescence, DNA damage and repair, and control of the cell cycle (24). It has also been reported that FOXM1 was overexpressed in a variety of human cancers and that the oncogenic potential of this gene is based on its ability to reactivate target genes involved in different stages of cancer development (25). It has been shown that the positive feedback of FOXM1 promotes the growth and invasion of gastric cancer and that FOXM1 promotes gastric cancer progression by interacting with PVT1 (26). FOXM1 has also been reported in non-serious epithelial ovarian carcinoma: FOXM1 was upregulated in all epithelial ovarian cancers (27). It has also been shown that the FOXM1-PSMB4 axis can play a catalytic role in the proliferation and development of cervical cancer (28). More surprisingly, FOXM1 plays a vital role in many other cancers (29–32). In our study, FOXM1 was upregulated in tumor tissues compared to normal tissues, suggesting a significant correlation with melanoma. Previous studies have shown that higher levels of FOXM1 in tumor tissues have been strongly associated with low prognosis in melanoma patients, consistent with our study (33–35). Kinesin Family Member 20A (KIF20A) is also a protein-encoding gene, and what is known about the diseases associated with this gene is mainly familial restriction Familial Isolated Restrictive Cardiomyopathy and Charcot- Marie-Tooth Disease, Type 4C. Research has also been conducted on the role of this gene in cancer. It has been reported that patients with bladder cancer with high expression of KIF20A have poorer tumor stages and that KIF20A promotes metastasis and proliferation of bladder cancer cells (36). Also, it has been shown that skin tumor thickness in KIF20A-positive patients with primary melanoma is significantly greater than skin tumor thickness in patients negative for this gene and that KIF20A-positive patients are more likely to relapse earlier (37). It is well known that while tumor recurrence has a very significant relationship with patient prognosis, this indirectly suggests that KIF20A is associated with survival in melanoma patients, which is consistent with the results of our study. The TPX2 Microtubule Nucleation Factor (TPX2) is a protein-coding gene. The main diseases known to be associated with the TPX2 gene include Capillary Leak Syndrome and Colorectal Cancer. It has been shown that activation of TPX2 expression increases the invasion and proliferation of cervical cancer, promoting cancer development (38). A study of TPX2 in esophageal cancer showed that the 5-year survival rate of esophageal cancer patients with concomitant high TPX2 expression levels was significantly lower than that of esophageal cancer patients with low TPX2 expression levels (39). Interestingly, in our study, patients with high TPX2 expression of melanoma had a relatively shorter overall survival than patients with low expression. Cell Division Cycle 20 (CDC20) is also a protein-coding gene. The main diseases known to be associated with this gene are Ceroid Lipofuscinosis, Neuronal, 2. Back in 2015, there were reports that CDC20 could be used as a novel cancer treatment modality (40). In hepatocellular carcinoma, the upregulation of CDC20 expression predicted a decline in overall survival and disease-free survival (41). Exonuclease 1 (EXO1) is a protein-coding gene. Diseases associated with EXO1 include Werner’s syndrome and Aicardi-Goutieres syndrome. In a recent article on the regulation of bladder cancer cells by phospholipase C-ϵ through EXO1, the authors noted that gene expression of EXO1 was significantly higher in 72 bladder cancer tissue specimens than in 24 adjacent paracancerous tissue samples (42). These five genes have been well-reported in other cancers, and there is not enough evidence to confirm their role in melanoma. In summary, the expression of the five hub genes we studied were all strongly associated with cancer, and in our study, high levels of expression of these genes were accompanied by shorter survival times for melanoma patients.

Our study combines the WGCNA approach with the DEGs approach through bioinformatics, searching for Hub genes through CytoHubba, a Cytoscape plugin, performing GO enrichment analysis and KEGG pathway analysis of the resulting intersection genes, as well as gene expression of different genes in the metastatic and primary tumor groups, and for the top 10 hub genes Survival analysis and prediction of disease-free survival with the GEPIA database were performed. Finally, the accuracy of our analysis was validated by immunohistochemistry experiments. The protein expression of FOXM1, KIF20A, TPX2, CDC20, and EXO1 was higher in melanoma than in paraneoplastic tissues, consistent with our analysis results. Our study, like others, has limitations regarding the different tumor types. Although we identified potential prognostic genes between melanoma and normal tissue using three different sources of databases with two different bioinformatics analyses, it was less accurate for each of the different subtypes of melanoma patients. Besides, this study should have done more adequate experiments to verify the role of the genes derived from our analysis in melanoma.

In conclusion, by combining the WGCNA analysis method with differentially expressed gene analysis, our study identified the genes FOXM1, KIF20A, TPX2, CDC20, and EXO1 highly correlated with survival melanoma patients and have the potential to serve as a prognostic biomarker in melanoma. Finally, we verified the accuracy and feasibility of our analysis results through immunohistochemistry experiments.

## Conclusion

FOXM1, KIF20A, TPX2, CDC20, and EXO1 are hub genes of melanoma prognostic, and their high expression is strongly associated with low prognosis in melanoma patients. FOXM1, KIF20A, TPX2, CDC20, and EXO1 could be used as biomarkers for melanoma diagnosis, treatment, and prognosis prediction.

## Data Availability Statement

The original contributions presented in the study are included in the article/supplementary material. Further inquiries can be directed to the corresponding author.

## Ethics Statement

The studies involving human participants were reviewed and approved by Ethical Review Committee of the First Clinical Affiliated Hospital of Guangxi Medical University. Written informed consent for participation was not required for this study in accordance with the national legislation and the institutional requirements.

## Author Contributions

JJ, CL, and XZ designed the study. GX, TL, SL, and CY analyzed the data. ZZ, ZL, ZW, JC, TC, and HL visualized the figures. JJ wrote and revised the manuscript. CL and XZ revised the manuscript. All co-authors participated in the laboratory operations. All authors contributed to the article and approved the submitted version.

## Funding

This study was supported by the Youth Science Foundation of Guangxi Medical University, Grant/Award Numbers: GXMUYFY201712; Guangxi Young and Middle-aged Teacher’s Basic Ability Promoting Project, Grant/Award Number: 2019KY0119; and National Natural Science Foundation of China, Grant/Award Numbers: 81560359, 81860393.

## Conflict of Interest

The authors declare that the research was conducted in the absence of any commercial or financial relationships that could be construed as a potential conflict of interest.

## Abbreviations

WGCNA, Weighted gene co-expression network analysis; DEGs, Differentially expressed genes; PPI, protein-protein functional interaction network; GO, Gene Ontology; CC, Cellular component; BP, Biological process; MF, Molecular function; OS, overall survival.

## References

[1] Rastrelli M Tropea S Rossi CR Alaibac M. Melanoma: epidemiology, risk factors, pathogenesis, diagnosis and classification In Vivo. In Vivo (2014) 28:1005–11. 25398793

[2] Pavri SN Clune J Ariyan S Narayan D. Malignant Melanoma: Beyond the Basics. Plast Reconstr Surg (2016) 138:330e–40e. 10.1097/PRS.0000000000002367 27465194

[3] Hanson J Demer A Liszewski W Foman N Maher I. Improved overall survival of melanoma of the head and neck treated with Mohs micrographic surgery versus wide local excision. J Am Acad Dermatol (2020) 82(1):149–55. 10.1016/j.jaad.2019.08.059 31473297

[4] Best MG Wesseling P Wurdinger T. Tumor-Educated Platelets as a Noninvasive Biomarker Source for Cancer Detection and Progression Monitoring. Cancer Res (2018) 78:3407–12. 10.1158/0008-5472.CAN-18-0887 29921699

[5] Chu H-W Chang K-P Hsu C-W Chang IY-F Liu H-P Chen Y-T. Identification of Salivary Biomarkers for Oral Cancer Detection with Untargeted and Targeted Quantitative Proteomics Approaches. Mol Cell Proteomics (2019) 18:1796–806. 10.1074/mcp.RA119.001530 PMC6731081 31253657

[6] Shan C Zhang Y Hao X Gao J Chen X Wang K. Biogenesis, functions and clinical significance of circRNAs in gastric cancer. Mol Cancer (2019) 18:136. 10.1186/s12943-019-1069-0 31519189 PMC6743094

[7] Langfelder P Horvath S. WGCNA: an R package for weighted correlation network analysis. BMC Bioinf (2008) 9:559. 10.1186/1471-2105-9-559 PMC2631488 19114008

[8] Unfried JP Serrano G Suárez B Sangro P Ferretti V Prior C. Identification of Coding and Long Noncoding RNAs Differentially Expressed in Tumors and Preferentially Expressed in Healthy Tissues. Cancer Res (2019) 79(20):5167–80. 10.1158/0008-5472.CAN-19-0400 31387921

[9] Ma Q Xu Y Liao H. Identification and validation of key genes associated with non-small-cell lung cancer. J Cell Physiol (2019) 234:22742–52. 10.1002/jcp.28839 31127628

[10] Grossi E Raimondi I Goñi E González J Marchese FP Chapaprieta V. A lncRNA-SWI/SNF complex crosstalk controls transcriptional activation at specific promoter regions. Nat Commun (2020) 11(1):936. 10.1038/s41467-020-14623-3 32071317 PMC7028943

[11] San Segundo-Val I. Sanz-Lozano CS. Introduction to the Gene Expression Analysis Methods. Mol Biol (2016) 1434:29–43. 10.1007/978-1-4939-3652-6_3 27300529

[12] Talantov D Mazumder A. Yu JX Briggs T Jiang Y Backus J. Novel genes associated with malignant melanoma but not benign melanocytic lesions. Clin Cancer Res (2005) 11:7234–42. 10.1158/1078-0432.CCR-05-0683 16243793

[13] Ritchie ME Phipson B Wu Di Hu Y. Law CW Shi W. limma powers differential expression analyses for RNA-sequencing and microarray studies. Nucleic Acids Res (2015) 43:e47. 10.1093/nar/gkv007 25605792 PMC4402510

[14] Chen H. Boutros PC. VennDiagram: a package for the generation of highly-customizable Venn and Euler diagrams in R BMC. bioinformatics (2011) 12:35. 10.1186/1471-2105-12-35 21269502 PMC3041657

[15] Gaudet P Dessimoz C. Gene Ontology: Pitfalls, Biases, and Remedies. Methods Mol Biol (2017) 1446:189–205. 10.1007/978-1-4939-3743-1_14 27812944

[16] Kanehisa M Furumichi M Tanabe M Sato Y Morishima K. KEGG: new perspectives on genomes, pathways, diseases and drugs. Nucleic Acids Res (2017) 45:D353–D61. 10.1093/nar/gkw1092 PMC5210567 27899662

[17] Yu G Wang L-G Han Y He Q-Y. clusterProfiler: an R package for comparing biological themes among gene clusters. OMICS (2012) 16:284–87. 10.1089/omi.2011.0118 PMC3339379 22455463

[18] Szklarczyk D Morris JH Cook H Kuhn M Wyder S Simonovic M. The STRING database in 2017: quality-controlled protein-protein association networks, made broadly accessible. Nucleic Acids Res (2017) 45:D362–8. 10.1093/nar/gkw937 PMC5210637 27924014

[19] Shannon P Markiel A Ozier O Baliga NS Wang JT Ramage D. Cytoscape: a software environment for integrated models of biomolecular interaction networks. Genome Res (2003) 13:(11)2498–504. 10.1101/gr.1239303 PMC403769 14597658

[20] Mantovani A Cassatella MA Costantini C Jaillon S. Neutrophils in the activation and regulation of innate and adaptive immunity. Nat Rev Immunol (2011) 11:519–31. 10.1038/nri3024 21785456

[21] Biton M Haber AL Rogel N Burgin G Beyaz S Schnell A. T Helper Cell Cytokines Modulate Intestinal Stem Cell Renewal and Differentiation. Cell (2018) 175(5):1307–20 .e22. 10.1016/j.cell.2018.10.008 30392957 PMC6239889

[22] Dror S Sander L Schwartz H Sheinboim D Barzilai A Dishon Y. Melanoma miRNA trafficking controls tumour primary niche formation. Nat Cell Biol (2016) 18(9):1006–17. 10.1038/ncb3399 27548915

[23] Lee TI Young RA. Transcriptional regulation and its misregulation in disease. Cell (2013) 152:1237–51. 10.1016/j.cell.2013.02.014 PMC3640494 23498934

[24] Yao S Fan LY Lam EW. The FOXO3-FOXM1 axis: A key cancer drug target and a modulator of cancer drug resistance. Semin Cancer Biol (2018) 50:77–89. 10.1016/j.semcancer.2017.11.018 29180117 PMC6565931

[25] Gartel AL. FOXM1 in Cancer: Interactions and Vulnerabilities. Cancer Res (2017) 77:3135–39. 10.1158/0008-5472.CAN-16-3566 PMC5519300 28584182

[26] Xu MD Wang Y Weng W Wei P Qi P Zhang Q. PVT1A Positive Feedback Loop of lncRNA- and FOXM1 Facilitates Gastric Cancer Growth and Invasion. Clin Cancer Res (2017) 23:(8)2071–80. 10.1158/1078-0432.CCR-16-0742 27756785

[27] Tassi RA Todeschini P Siegel ER. FOXM1 expression is significantly associated with chemotherapy resistance and adverse prognosis in non-serous epithelial ovarian cancer patients. (2017) 36:63. 10.1186/s13046-017-0536-y PMC5422964 28482906

[28] Zhou DM Liu J Liu F Luo GW Li HT Zhang R. A novel FoxM1-PSMB4 axis contributes to proliferation and progression of cervical cancer. Biochem Biophys Res Commun (2020) 521(3):746–52. 10.1016/j.bbrc.2019.10.183 31699366

[29] Li XY Wu HY Mao XF Jiang LX Wang YX. USP5 promotes tumorigenesis and progression of pancreatic cancer by stabilizing FoxM1 protein. Biochem Biophys Res Commun (2017) 492:48–54. 10.1016/j.bbrc.2017.08.040 28807830

[30] Cui J Shi M Xie D Wei D Jia Z Zheng S. FOXM1 promotes the warburg effect and pancreatic cancer progression via transactivation of LDHA expression. Clin Cancer Res (2014) 20(10):2595–606. 10.1158/1078-0432.CCR-13-2407 PMC4024335 24634381

[31] Zhou Z Chen H Xie R Wang H Li S Xu Q. Epigenetically modulated FOXM1 suppresses dendritic cell maturation in pancreatic cancer and colon cancer. Mol Oncol (2019) 13(4):873–93. 10.1002/1878-0261.12443 PMC6441919 30628173

[32] Arceci A Bonacci T Wang X Stewart K Damrauer JS Hoadley KA. FOXM1 Deubiquitination by USP21 Regulates Cell Cycle Progression and Paclitaxel Sensitivity in Basal-like Breast Cancer. Cell Rep (2019) 26:3076–86.e6. 10.1016/j.celrep.2019.02.054 30865895 PMC6425951

[33] Puig-Butille JA Vinyals A Ferreres JR Aguilera P Cabré E Tell-Martí G. AURKA Overexpression Is Driven by FOXM1 and MAPK/ERK Activation in Melanoma Cells Harboring BRAF or NRAS Mutations: Impact on Melanoma Prognosis and Therapy. J Invest Dermatol (2017) 137(6):1297–310. 10.1016/j.jid.2017.01.021 28188776

[34] Kruiswijk F Hasenfuss SC Sivapatham R Baar MP Putavet D Naipal KA. Targeted inhibition of metastatic melanoma through interference with Pin1-FOXM1. signaling (2016) 35(17):2166–77. 10.1038/onc.2015.282 PMC4757516 26279295

[35] Kuzu OF Gowda R Sharma A Noory MA Kardos G Madhunapantula SV. Identification of WEE1 as a target to make AKT inhibition more effective in melanoma. Cancer Biol Ther (2018) 19(1):53–62. 10.1080/15384047.2017.1360446 28853983 PMC5790369

[36] Shen T Yang L Zhang Z Yu J Dai L Gao M. KIF20A Affects the Prognosis of Bladder Cancer by Promoting the Proliferation and Metastasis of Bladder Cancer Cells. Dis Markers (2019) 2019:4863182. 10.1155/2019/4863182 31093305 PMC6481133

[37] Yamashita J Fukushima S Jinnin M Honda N Makino K Sakai K. Kinesin family member 20A is a novel melanoma-associated antigen. Acta Derm Venereol (2012) 92(6):593–7. 10.2340/00015555-1416 22854760

[38] Song T Xu A Zhang Z Gao F Zhao L Chen X. CircRNA hsa_circRNA_101996 increases cervical cancer proliferation and invasion through activating TPX2 expression by restraining miR-8075. J Cell Physiol (2019) 234:14296–305. 10.1002/jcp.28128 PMC13483104 30633364

[39] Sui C Song Z Yu H Wang HJ. Prognostic significance of TPX2 and NIBP in esophageal cancer;. Oncol Lett (2019) 18:4221–29. 10.3892/ol.2019.10747 PMC6732995 31516617

[40] Wang L Zhang J Wan L Zhou X Wang Z Wei WJ. Targeting Cdc20 as a novel cancer therapeutic strategy. Pharmacol Ther (2015) 151:141–51. 10.1016/j.pharmthera.2015.04.002 PMC4457591 25850036

[41] Zhuang L Yang Z Meng ZJ. Upregulation of BUB1B, CCNB1, CDC7, CDC20, and MCM3 in Tumor Tissues Predicted Worse Overall Survival and Disease-Free Survival in Hepatocellular Carcinoma Patients. BioMed Res Int (2018) 2018:7897346. 10.1155/2018/7897346 30363964 PMC6186344

[42] Fan J Zhao Y Yuan H Yang J Li T He Z. Phospholipase C-ϵ regulates bladder cancer cells via ATM/EXO1. Am J Cancer Res (2020) 10:2319–36. PMC7471350 32905533

